# Patient visits and prescriptions for attention-deficit/hyperactivity disorder from 2017–2021: Impacts of COVID-19 pandemic in primary care

**DOI:** 10.1371/journal.pone.0281307

**Published:** 2023-03-13

**Authors:** Debra A. Butt, Ellen Stephenson, Sumeet Kalia, Rahim Moineddin, Karen Tu

**Affiliations:** 1 Department of Family and Community Medicine, Scarborough General Hospital, Scarborough Health Network, Toronto, Ontario, Canada; 2 Department of Family and Community Medicine, Temerty Faculty of Medicine, University of Toronto, Toronto, Ontario, Canada; 3 Research and Innovation, Department of Family and Community Medicine, North York General Hospital, Toronto, Ontario, Canada; 4 Toronto Western Family Health Team, University Health Network, Toronto, Ontario, Canada; Universitair Kinderziekenhuis Koningin Fabiola: Hopital Universitaire des Enfants Reine Fabiola, BELGIUM

## Abstract

**Objective:**

To determine whether more patients presented with Attention-deficit/hyperactivity disorder (ADHD)-related visits and/or sought care from family physicians more frequently during the COVID-19 pandemic.

**Methods:**

Electronic medical records from the University of Toronto Practice-Based Research Network were used to characterize changes in family physician visits and prescriptions for ADHD medications. Annual patient prevalence and visit rates pre-pandemic (2017–2019) were used to calculate the expected rates in 2020 and 2021. The expected and observed rates were compared to identify any pandemic-related changes.

**Results:**

The number of patients presenting for ADHD-related visits during the pandemic was consistent with pre-pandemic trends. However, observed ADHD-related visits in 2021 were 1.32 times higher than expected (95% CI: 1.05–1.75), suggesting that patients visited family physicians more frequently than before the pandemic.

**Conclusion:**

Demand for primary care services related to ADHD has continued to increase during the pandemic, with increased health service use among those accessing care.

## Introduction

The COVID-19 pandemic has disrupted the daily lives of people living in Ontario, Canada and worldwide. On March 17, 2020, Ontario declared a state of emergency due to COVID-19. From March 14, 2020 to May 15, 2021, Ontario schools were closed for 20 weeks in total, much longer than any other Canadian province or territory [1]. From February 16, 2020 to October 31, 2021, Canada has had the longest duration of primary and secondary school closures at 51 weeks, second only to the United States, but higher than other Group of 10 (G10) countries like Belgium, France, Germany, Italy, Japan, the Netherlands, Sweden, Switzerland, and the United Kingdom [2]. Canada was also the only country in the G10 to maintain a sustained moderate to severe degree of restrictions on internal movement, cancellation of public events, public gatherings, education and workplace closures and international travel since the spring of 2020 [2]. These multiple lockdowns affecting all ages resulted in remote learning and working from home as part of the social distancing requirement to prevent the viral transmission of COVID-19 and significantly impacted the mental health of the general population especially those with a pre-existing condition of Attention-deficit/hyperactivity disorder (ADHD) [3].

Evidence from international systematic reviews and/or meta-analyses in the general population during the COVID-19 pandemic has consistently demonstrated that the general population experienced mental health symptoms [4–9]. A systemic review of 30 studies assessing the mental health of children ≤12 years of age between February 2020 and July 2021 demonstrated more mental health symptoms during the pandemic [5]. A rapid systematic review of 80 studies (until March 29, 2020) from 11 countries involving children and youth (N = 51,576 participants; mean age 15.3 years) demonstrated that healthy children and adolescents are more likely to experience high rates of depression and anxiety during and after enforced isolation, where the duration and not the intensity of loneliness is more strongly correlated with mental health symptoms [4]. Similarly, a systematic review of 36 studies from 11 countries involving 79,781 children and adolescents during the first wave of the COVID-19 pandemic (February to July 2020) found that school closures as part of the social lockdown measures resulted in poorer mental health and well-being among children and adolescents [6]. These findings in children and youth were also consistent with adults. Systematic reviews and meta-analyses in healthy adults have also indicated higher rates of anxiety, depression, post-traumatic stress disorder, psychological distress, and stress during the COVID-19 pandemic [7–10]. A large Canadian cross-sectional study on mental health found that children and adolescents without and with psychiatric diagnoses tended to deteriorate during the first wave of the COVID-19 pandemic with a higher rate of deterioration of 40–56% in those with a pre-existing attention disorder [3]. Those living with attention disorders have a difficult time adjusting to profound changes in routine, especially with education and employment, and can be adversely affected by social isolation and COVID disease containment measures more so than the general population [11–15].

ADHD is a childhood-onset neurodevelopmental disorder and one of the most common childhood psychiatric conditions in Canada [11, 16]. ADHD is a chronic disorder with developmentally inappropriate symptoms of inattention, hyperactivity and/or impulsivity that can lead to impairments in many aspects of life. This disorder is more common in males than females. Although ADHD symptoms decrease with age, evidence suggests that over 90% of children diagnosed with ADHD will continue to struggle with residual and sometimes fluctuating symptoms and impairments into adulthood [17]. The greater than 2:1 ratio of an ADHD diagnosis in males to females seen in childhood tends to decrease to a 1:1 ratio in adulthood [18]. The comorbidity rate is also higher in adults where as many as 80% of adults with ADHD are reported to have at least one comorbid psychiatric disorder [19, 20] which can adversely affect patient prognosis [21]. A qualitative meta-synthesis of 7 qualitative studies of adults, newly diagnosed with ADHD in adulthood, demonstrated that delayed diagnosis caused suffering and dysfunction that could have been lessened through earlier detection and management [22].

Several studies have reported on the worsening of ADHD symptoms during the COVID-19 pandemic. A Canadian online survey to caregivers of children with ADHD aged 5–18 years (n = 587 surveys) revealed that during the COVID pandemic, parents reported that their children with ADHD experienced greater ADHD symptoms, depression and anxiety [13]. This finding of worsening ADHD symptoms in children diagnosed with ADHD during the COVID pandemic is consistent with other international studies from China [23] and France [24]. A systematic review of 12 studies (before February 25, 2021) from 9 countries involving 3,028 participants aged 4–27 years diagnosed with ADHD reported increased ADHD symptoms and psychological difficulties during the COVID pandemic [15].

For most Canadians seeking healthcare for mental health conditions, including ADHD, their family physician is the first point of contact. The Canadian Paediatric Society (CPS), American Academy of Pediatrics (AAP) and Canadian ADHD Resource Alliance (CADDRA) have all stated that ADHD care including diagnosis and management is within the practice of family physicians [25–27]. A cross-sectional retrospective study conducted in children and youth aged 1–24 years in Ontario, Canada demonstrated that those individuals diagnosed with ADHD had significantly more visits to family doctors for mental health-related concerns compared to non-ADHD patients [28]. In recent decades, there has been an increase in the prevalence of ADHD medication prescribing in all age groups in Canadian family medicine practices [29]. Management for ADHD may also include referral to specialists and/or other health care providers as family physicians are the gatekeepers in the Canadian primary care setting and patients and their families cannot access specialists directly [30] unless they seek private non-insured services.

We observed an increase in patient inquires into ADHD across the lifespan in family medicine practices in Ontario, Canada during the COVID-19 pandemic, so we set out to examine the trend of patient visits and prescriptions for ADHD prior to (2017–2019) and during the pandemic (2020–2021). We aimed to determine the extent to which this increase was the result of a greater number of visits per patient presenting with ADHD as the most responsible reason for the visit to the primary care physician and/or an increase in the number of patients presenting for ADHD-related visits. Our research questions are: 1) did health service use for ADHD increase in the primary care setting during the COVID-19 pandemic, and/or 2) did the prevalence of ADHD-related visits increase during the COVID-19 pandemic?

## Methods

### Study design and setting

We conducted a retrospective study of family medicine patients in the University of Toronto Practice-Based Research Network (UTOPIAN) from January 1, 2017-December 31, 2021. We used data from the UTOPIAN Data Safe Haven (dfcm.utoronto.ca/utopian-data-safe-haven), a primary care EMR database with records collected from family physician practices in the Greater Toronto Area and beyond in Ontario, Canada. Records that meet minimum data quality criteria [31] are available for use in research studies. The Ontario government introduced new billing codes in response to the COVID-19 pandemic to allow family physicians to offer care virtually via telephone or video [32, 33]. Prior to this policy change, the use of virtual care in Ontario was extremely limited [33, 34].

### Eligibility criteria

Eligibility was assessed at the end of each calendar year, recognizing that some patients will join and leave a family medicine practice over time. Patients were enrolled in the cohort based on the following criteria: 1) they were registered to a physician for whom the data extracted met minimum quality criteria, 2) they had age and sex recorded in the EMR, 3) they had at least one billing code for an office or virtual visit with a family physician [32, 35, 36] within the past 5 years, 4) they were aged 5–55 years, and 5) at least 1 year of data was recorded in the EMR (i.e., 1 or more years since their first visit). Patients were excluded five years after their most recent visits. We selected the age range from 5–55 years because very few visits or prescriptions related to ADHD occurred among patients outside this range.

### Outcome measures

We assessed primary care health utilizations for ADHD using three outcome measures: 1) total number of ADHD visits with Ontario Health Insurance Program (OHIP) billing code 314 for ADHD and the International Classification of Diseases Ninth revision (ICD-9) code also uses 314 for ADHD, per 1000 patients as a measure of service demand on the health care system; 2) total number of patients with at least one billing for a visit (S1 Appendix) with a diagnostic code for ADHD annually; and 3) a prescription for a medication used to treat ADHD (S1 Table). Outcome measure (1) was used to quantify changes in healthcare utilization for ADHD-related visits each year. We used outcome measures (2) and (3) to describe the annual patient prevalence for both ADHD-related visits and medication prescriptions, respectively. Increases in the visit rate could result from an increase in patient prevalence and/or an increase in the intensity of visits for each patient presenting in primary care. This approach allowed us to assess both possibilities.

### Sociodemographic measures

The UTOPIAN database includes measures of demographic characteristics (sex, age, neighborhood income quintile, rurality). Neighborhood-level income quintiles and rurality were derived based on the patient’s residential postal codes using Statistics Canada’s Postal Code Conversion Files [37].

### Statistical analysis

We employed a repeated cross-sectional design to describe the annual visit and patient prevalence rates from January 1, 2017- December 31, 2021. To evaluate whether the COVID-19 pandemic had a significant impact on primary care services for ADHD, we used data from the years before the pandemic (2017–2019) to predict the expected rates in 2020 and 2021 during the pandemic. We used Poisson regression with exchangeable correlation structure to account for the clustering of count data. In particular, the Poisson regression was used to estimate the expected count for i) total number of visits for ADHD, ii) total number of patients with at least one ADHD-related visit, and iii) the total number of patients with at least one ADHD-specific medication prescribed each year. The log of total number of patients enrolled in the cohort each year was used as an offset term. We hypothesized that the prevalence of ADHD differs by age and sex, and we performed subgroup analyses using Poisson regression by stratifying for age group and sex, while adjusting for income quintiles and rurality. The expected counts in 2020 and 2021 were reported using the sub-group analysis (i.e. stratified Poisson regression). We compared the expected and observed counts in 2020 and 2021 using two-sided z-test with statistical significance evaluated at 5% alpha-level and reported 95% confidence intervals (CI). Statistical analyses were performed using SAS software version 9.4.

### Ethics approval

This study was approved by the Research Ethics Boards of the University of Toronto (#40129) and North York General Hospital (#20–0044). Physicians in this study provided written informed consent to have their EMR data extracted, de-identified, and used for research purposes; patients can opt out of uses for research.

## Results

### Sample characteristics

A total of 266,208 patients met criteria for inclusion (206,369 to 221,519 annually; Table 1).

**Table 1:** Sample characteristics.

	2017		2018		2019		2020		2021	
	N	%	N	%	N	%	N	%	N	%
**Age group (years)**										
05–09	15781	7.6	16364	7.6	16597	7.6	16677	7.5	16545	7.5
10–14	15230	7.4	15885	7.4	16247	7.5	16498	7.4	16141	7.3
15–19	17136	8.3	17740	8.3	18201	8.4	18396	8.3	18136	8.2
20–24	19690	9.5	20423	9.5	20746	9.5	20954	9.5	20318	9.2
25–34	42998	20.8	44863	21.0	45779	21.0	47088	21.3	46948	21.3
35–55	95534	46.3	98735	46.1	100321	46.0	101906	46.0	102121	46.4
**Sex**										
Female	113700	55.1	117577	54.9	119389	54.8	121636	54.9	121130	55.0
Male	92669	44.9	96433	45.1	98502	45.2	99883	45.1	99079	45.0
**Income Quintiles**										
Lowest	35886	17.4	38219	17.9	39211	18.0	40279	18.2	39945	18.1
Low-mid	31662	15.3	32888	15.4	33549	15.4	34377	15.5	34183	15.5
Middle	33455	16.2	34582	16.2	35098	16.1	35805	16.2	35620	16.2
Mid-high	41496	20.1	42808	20.0	43584	20.0	44273	20.0	43991	20.0
Highest	55571	26.9	56916	26.6	57733	26.5	58235	26.3	57680	26.2
Missing	8299	4.0	8597	4.0	8716	4.0	8550	3.9	8790	4.0
**Rurality**										
Urban	184321	89.3	191592	89.5	195452	89.7	199481	90.1	198241	90.0
Rural	15937	7.7	16055	7.5	16002	7.3	15827	7.1	15500	7.0
Missing	6111	3.0	6363	3.0	6437	3.0	6211	2.8	6468	2.9
**Total**	206369	100.0	214010	100.0	217891	100.0	221519	100.0	220209	100.0

Note: This study used an open cohort design such that patients could join and leave the cohort over time based on specified eligibility criteria. The decrease in sample size from 2020 to 2021 suggests that the rate at which patients were censored from the cohort was larger than the rate at which new patients were added. During the pandemic, many family physicians may have stopped accepting new patients and focused on supporting their existing patients.

Demographic characteristics were consistent with the UTOPIAN patient population and those most likely to access primary care services [36]. There were more females than males and more patients from higher income and urban neighborhoods. The age and sex composition of patients remained relatively stable across the 5-year study period. We noticed that the proportion of patients residing in rural regions decreased from 7.7% in 2017 to 7.0% in 2021 (Table 1).

### Visits for ADHD

From 2017 to 2021, 6961 visits for ADHD were observed. The visit rate per 1000 patients increased from 3.7 (772 visits for 206,369 patients) in 2017 to 5.4 (1177 visits for 217,891 patients) in 2019 (Table 2). Based on the observed data from 2017–2019, the expected rate was 6.51; 95% CI: 4.95–8.06 for 2020 and 7.97; 95% CI: 5.99–9.95 for 2021.

**Table 2:** Annual ADHD-related visit rates by age, sex, income quintile, and rurality.

	year									
	2017		2018		2019		2020		2021	
	Visits*	per 1000 patients	Visits*	per 1000 patients	Visits*	per 1000 patients	Visits*	per 1000 patients	Visits*	per 1000 patients
**Age group (years)**										
05–09	158	10.0	153	9.3	191	11.5	182	10.9	208	12.6
10–14	120	7.9	136	8.6	181	11.1	192	11.6	246	15.2
15–19	149	8.7	224	12.6	257	14.1	344	18.7	466	25.7
20–24	112	5.7	151	7.4	161	7.8	303	14.5	442	21.8
25–34	123	2.9	191	4.3	193	4.2	385	8.2	531	11.3
35–55	110	1.2	156	1.6	194	1.9	278	2.7	424	4.2
**Sex**										
Female	266	2.3	350	3.0	352	2.9	593	4.9	986	8.1
Male	506	5.5	661	6.9	825	8.4	1091	10.9	1331	13.4
**Income Quintiles**										
Lowest	123	3.4	120	3.1	149	3.8	175	4.3	265	6.6
Low-mid	78	2.5	137	4.2	161	4.8	264	7.7	360	10.5
Middle	106	3.2	138	4.0	165	4.7	253	7.1	383	10.8
Mid-high	175	4.2	229	5.3	295	6.8	373	8.4	496	11.3
Highest	273	4.9	372	6.5	374	6.5	583	10.0	762	13.2
Missing	17	2.0	15	1.7	33	3.8	36	4.2	51	5.8
**Rurality**										
Urban	687	3.7	903	4.7	1052	5.4	1576	7.9	2203	11.1
Rural	69	4.3	94	5.9	102	6.4	87	5.5	96	6.2
Missing	16	2.6	14	2.2	23	3.6	21	3.4	18	2.8
**Total**	772	3.7	1011	4.7	1177	5.4	1684	7.6	2317	10.5

*Total number of annual primary care visits (including in-person and virtual) with the diagnostic ICD-9 code 314 for ADHD.

**Fig 1:**
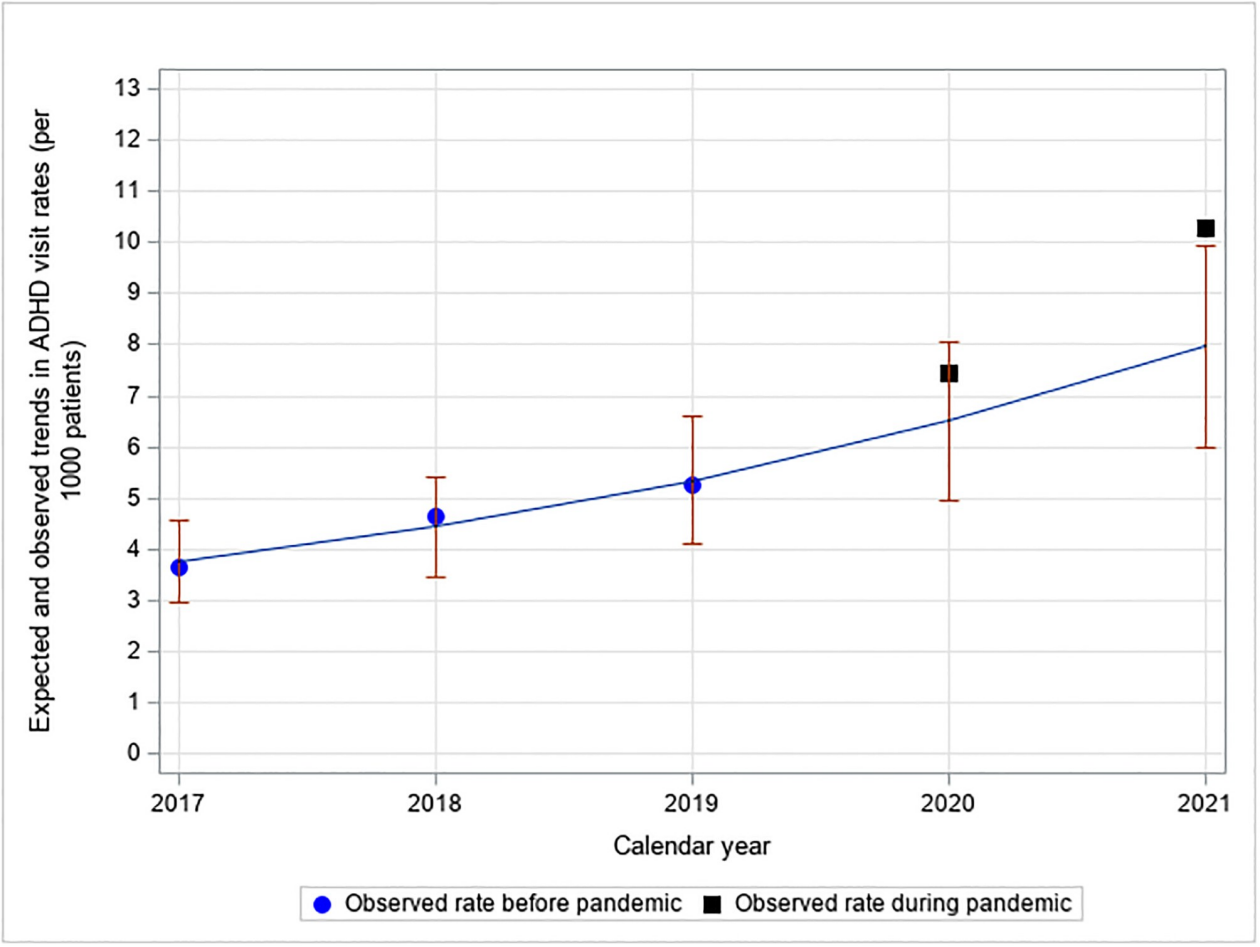
Expected and observed trends in ADHD-related visit rates during the COVID-19 pandemic*. *The pre-pandemic data (2017–2019) are used to estimate the expected rate during the pandemic years 2020–2021 (with 95% CI). Data for Fig 1 are found in the Supporting information file, see S2A Table in S2 Table.

Although the observed rate of 7.6 (1684/221,519) in 2020 was within the expected range, the observed rate of 10.5 (2317/220,209) in 2021 was 1.32; 95% CI: 1.05–1.75, times higher than expected (Fig 1). Furthermore, this significantly higher ADHD visit rate in 2021 was observed among the female age group 5–9 years old (ratio of observed to expected rate = observed/expected rate = 2.60; 95% CI: 1.53–8.85), among 20–24 year old females (observed/expected rate = 6.08; 95% CI: 3.30–38.27), among 25–34 year old females (observed/expected rate = 1.94; 95% CI: 1.12–7.18), among 35–55 year old females (observed/expected rate = 1.93; 95% CI: 1.12–6.92), and among 25–34 year old males (observed/expected rate = 1.97; 95% CI: 1.14–7.21) (S3 Table).

Despite the higher number of ADHD visits during the pandemic, the number of unique patients with at least one visit for ADHD per year remained consistent with pre-pandemic trends. The annual prevalence of patients visiting for ADHD increased steadily from 2.4 per 1000 patients in 2017 to 5.5 per 1000 patients in 2021 (Table 3).

**Table 3:** Annual prevalence of patients with one or more ADHD-related visits by age, sex, income quintile, and rurality.

	2017		2018		2019		2020		2021	
	N	per 1000 patients	N	per 1000 patients	N	per 1000 patients	N	per 1000 patients	N	per 1000 patients
**Age group (years)**										
05–09	99	6.3	109	6.7	123	7.4	115	6.9	124	7.5
10–14	78	5.1	92	5.8	120	7.4	123	7.5	147	9.1
15–19	97	5.7	136	7.7	170	9.3	178	9.7	242	13.3
20–24	74	3.8	108	5.3	117	5.6	174	8.3	228	11.2
25–34	85	2.0	113	2.5	123	2.7	191	4.1	258	5.5
35–55	70	0.7	95	1.0	120	1.2	140	1.4	208	2.0
**Sex**										
Female	187	1.6	228	1.9	244	2.0	330	2.7	512	4.2
Male	316	3.4	425	4.4	529	5.4	591	5.9	695	7.0
**Income Quintiles**										
Lowest	76	2.1	85	2.2	97	2.5	101	2.5	156	3.9
Low-mid	54	1.7	86	2.6	102	3.0	137	4.0	189	5.5
Middle	73	2.2	97	2.8	106	3.0	141	3.9	181	5.1
Mid-high	113	2.7	141	3.3	186	4.3	181	4.1	247	5.6
Highest	176	3.2	233	4.1	260	4.5	334	5.7	401	7.0
Missing	11	1.3	11	1.3	22	2.5	27	3.2	33	3.8
**Rurality**										
Urban	449	2.4	578	3.0	688	3.5	847	4.2	1132	5.7
Rural	45	2.8	67	4.2	69	4.3	58	3.7	61	3.9
Missing	9	1.5	8	1.3	16	2.5	16	2.6	14	2.2
**Total**	503	2.4	653	3.1	773	3.5	921	4.2	1207	5.5

**Fig 2:**
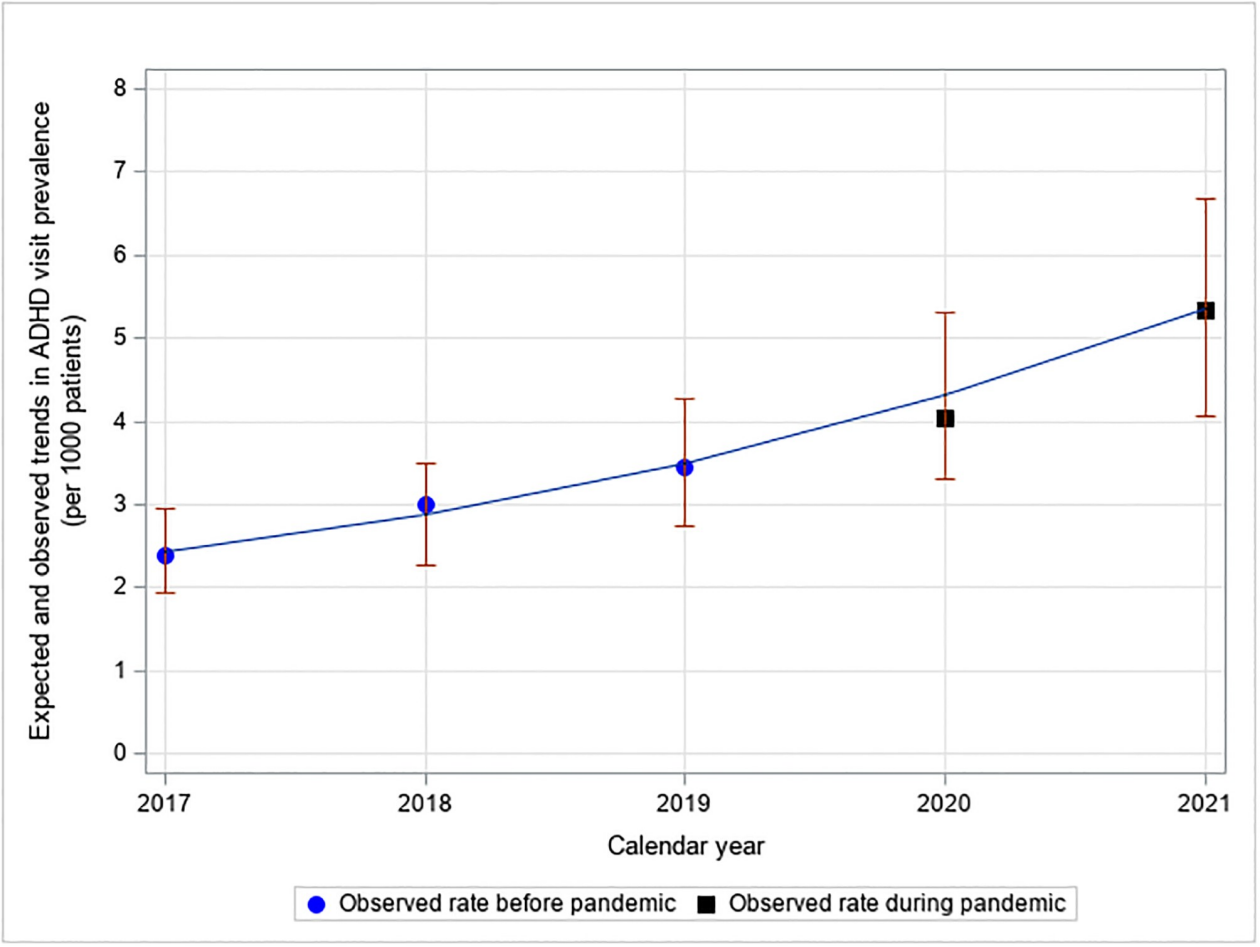
Expected and observed trends in ADHD-related visit prevalence during the COVID-19 pandemic*. *The pre-pandemic data (2017–2019) are used to estimate the expected rate during the pandemic years 2020–2021 (with 95% CI). Data for Fig 2 are found in the Supporting information file, see S2B Table in S2 Table.

The observed rates in 2020 and 2021 were within the expected range based on pre-pandemic trends (Fig 2), with similar patterns when data were stratified by age, sex, income, and rurality.

### Prescriptions for ADHD medications

In the longitudinal cohort (from 2017 to 2021), we found 56,224 unique prescriptions for ADHD medications. Of these, 19,206 (34.1%) occurred on the same date as a billing with a diagnostic code for ADHD; less specific diagnostic codes for behavior disorders of childhood and adolescence were also frequently used when prescribing these medications. We observed that the prescribing of ADHD medications on the same day as a patient visit for those aged 5–55 years of age was most associated with the OHIP billing code, 313 representing behavioural disorders of childhood and adolescence (21–24%) during the pre-pandemic period from 2017–2019 followed by the OHIP billing code 300 representing anxiety (19–20%) then 314 for ADHD (18–20%). During the pandemic period from 2020–2021, the most frequent OHIP billing code was 314 for ADHD (25%) followed by 313 for behavioural disorders of childhood and adolescence (22–24%) then 300 for anxiety (22–23%). We found a somewhat similar pattern of OHIP billing codes in the pre-pandemic period for children/adolescents. The most frequent OHIP billing code was 313 for behavioural disorders followed by 314 for ADHD and then 300 for anxiety, and this pattern was consistent during the pandemic period. For adults during the pre-pandemic period, the most frequent OHIP billing code was 300 for anxiety, followed by 313 for behavioural disorders then 314 for ADHD. During the pandemic period, the most frequently billed code for adults remained as 300 for anxiety followed by 314 for ADHD and then 313 for behavioural disorders. The annual prevalence of patients being prescribed medication for ADHD increased from 12.9 per 1000 patients in 2017 to 21.9 per 1000 patients in 2021 (Table 4).

**Table 4:** Annual prevalence of patients with one or more prescriptions for an ADHD-related medication by age, sex, income quintile, and rurality.

	2017		2018		2019		2020		2021	
	N	per 1000 patients	N	per 1000 patients	N	per 1000 patients	N	per 1000 patients	N	per 1000 patients
**Age group (years)**										
05–09	245	15.5	243	14.8	289	17.4	237	14.2	280	16.9
10–14	329	21.6	366	23.0	423	26.0	425	25.8	468	29.0
15–19	453	26.4	460	25.9	576	31.6	574	31.2	736	40.6
20–24	428	21.7	555	27.2	628	30.3	718	34.3	875	43.1
25–34	557	13.0	640	14.3	764	16.7	930	19.8	1235	26.3
35–55	650	6.8	784	7.9	908	9.1	1019	10.0	1222	12.0
**Sex**										
Female	1061	9.3	1218	10.4	1472	12.3	1700	14.0	2277	18.8
Male	1601	17.3	1830	19.0	2116	21.5	2203	22.1	2539	25.6
**Income Quintiles**										
Lowest	408	11.4	442	11.6	531	13.5	526	13.1	640	16.0
Low-mid	383	12.1	399	12.1	490	14.6	571	16.6	715	20.9
Middle	397	11.9	454	13.1	531	15.1	587	16.4	748	21.0
Mid-high	474	11.4	595	13.9	688	15.8	758	17.1	942	21.4
Highest	925	16.6	1077	18.9	1255	21.7	1354	23.3	1648	28.6
Missing	75	9.0	81	9.4	93	10.7	107	12.5	123	14.0
**Rurality**										
Urban	2399	13.0	2758	14.4	3257	16.7	3540	17.7	4413	22.3
Rural	212	13.3	241	15.0	273	17.1	297	18.8	336	21.7
Missing	51	8.3	49	7.7	58	9.0	66	10.6	67	10.4
**Total**	2662	12.9	3048	14.2	3588	16.5	3903	17.6	4816	21.9

**Fig 3:**
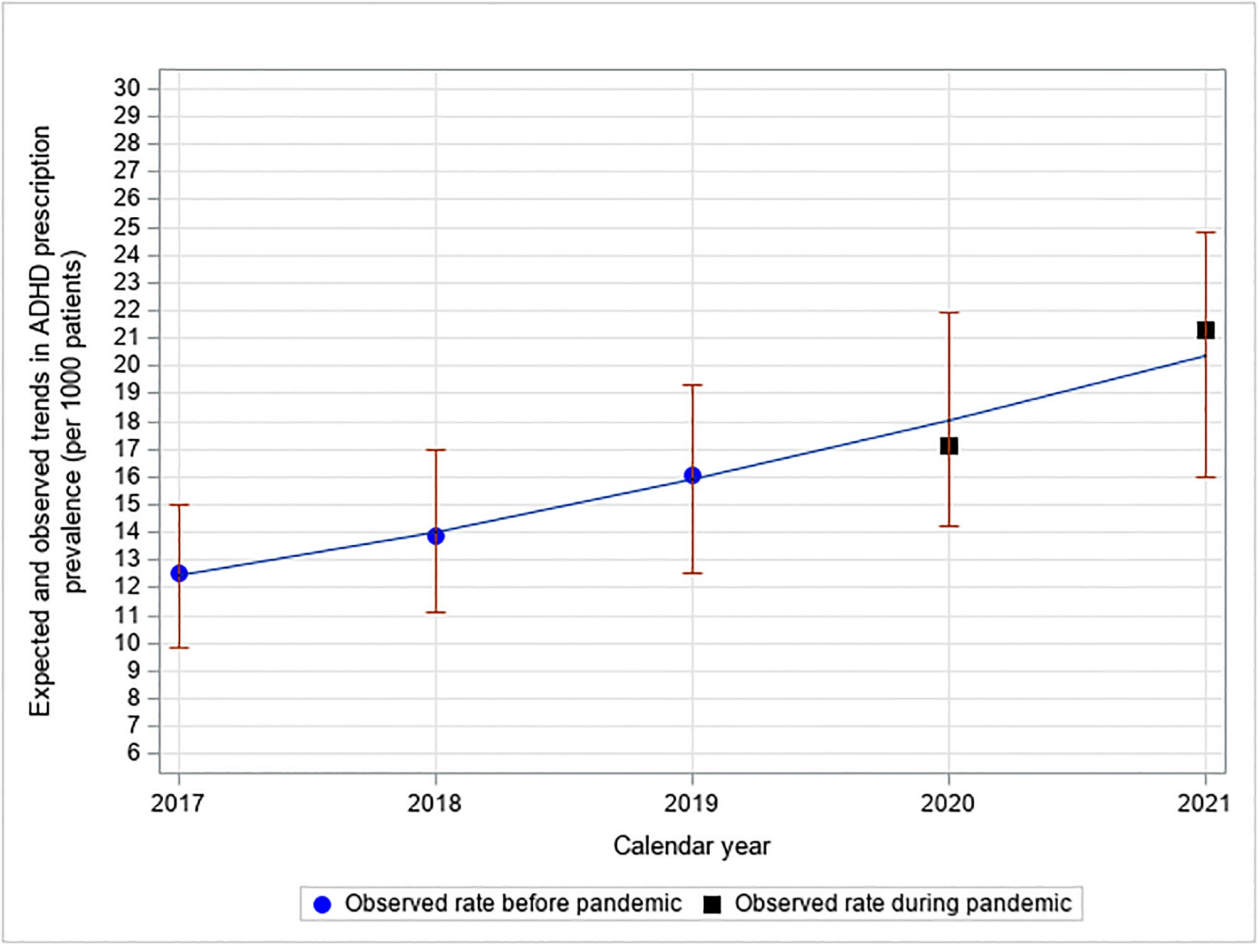
Expected and observed trends in ADHD prescription prevalence during the COVID-19 pandemic*. *The pre-pandemic data (2017–2019) are used to estimate the expected rate during the pandemic years 2020–2021 (with 95% CI). Data for Fig 3 are found in the Supporting information file, see S2C Table in S2 Table.

The prescription rates observed during the COVID-19 pandemic were within the expected range 2020 and 2021 based on pre-pandemic trends (Fig 3).

In general, male patients had a higher annual rate of ADHD-related visits (Table 2), ADHD-related visit prevalence (Table 3) and ADHD medications (Table 4) than females from 2017 to 2021. Patients residing in highest income quintiles had higher rates of ADHD-related visits, ADHD-related visit prevalence and ADHD medications than those residing in lowest income quintiles.

## Discussion

During the COVID-19 pandemic in 2021, we observed a 32% overall relative increase in the total number of visits to family physicians for ADHD for ages 5–55 years in Ontario, Canada compared to the expected number of visits. This was in addition to the growing demand for ADHD-related services that existed even before the pandemic. This reflects an increase in the intensity of health service utilization in the primary care setting for those seeking care related to ADHD. Similar findings have been observed for other mental health services related to anxiety and depression [36]. There was an increase in the number of visits per patient relative to before the pandemic. The introduction of virtual appointment via telephone or video may have reduced some barriers to accessing care and allowed for closer follow-up. However, it will be important for future research to examine impacts and value of virtual care for patients with ADHD. The increase in the intensity of health service utilization we observed is also consistent with the increase in ADHD symptom burden in the population [11–15]. People living with ADHD were experiencing more difficulties and this is reflected in an increased demand on family physicians to spend more time addressing ADHD.

We found that females in the age groups 5–9 years and those between 20–55 years visited their family physicians more frequently during the pandemic period in 2021 compared to the pre-pandemic period. In the younger age group, parents likely sought greater support from their family physicians for their female children related to ADHD and this could have stemmed from school closures, remote learning, and social isolation. Older females may have had greater responsibilities to cope with not only related to changes in education and learning during the pandemic but also with employment and parenting/family obligations which may have led to seeking more health care related to ADHD [38]. It may be that access to family physicians more frequently during the pandemic was easier due to the uptake of virtual care and marked decreases in other health care services like periodic health exams, well-baby visits and preventive care services [36]. In our study, we also found that patients residing in higher income quintiles had higher rates of ADHD-related visits, visit prevalence and ADHD medications than those residing in lower income quintiles which is consistent with evidence supporting an increased risk of diagnosing ADHD in high-income households [39, 40].

The number of people diagnosed with ADHD and prescribed medication to treat ADHD has increased in recent years. A retrospective cohort study involving the population of Quebec from 2000–2018 demonstrated a steady increase in prevalence of ADHD among children and young adults [41]. In addition, a Canadian study conducted from 2005–2015 using EMR data showed a 2.6-fold increase in the prevalence of prescribing ADHD medications to preschoolers, a 2.5-fold increase in school-aged children and a 4-fold increase in adults [29]. Consistent with these findings, we found that the number of patients visiting related to ADHD increased from 2.4 (per 1000 patients) in 2017 to 5.5 (per 1000 patients) in 2021 (Table 2), and similarly the number of patients being prescribed ADHD medication at least once annually increased from 12.9 (per 1000 patients) in 2017 to 21.9 (per 1000 patients) in 2021 (Table 3). A US retrospective cohort study involving children aged 6–17 years using electronic health records from primary care providers (mainly pediatricians) found that ADHD medication rates remained stable throughout the first year of the pandemic (March 15, 2020-March 15, 2021) [42]. We also found that ADHD medication prescribing patterns did not deviate significantly from pre-pandemic trends.

The early stages of the pandemic in 2020 did not necessarily coincide with an increase in mental health presentations to family physicians but the adaptation to the new conditions imposed by the COVID-19 pandemic increased workloads on the frontline of mental health services offered in the Canadian primary care setting [43]. Our study found that family physicians faced increased demand for mental health care services for ADHD in 2021. However, several studies have demonstrated that family physicians in North America are neither familiar with the diagnostic criteria for ADHD or use the criteria appropriately in their practices [16, 25, 26]. A US study found that ADHD management is not increasingly undertaken by recently trained pediatric primary care providers [42] and that pediatricians who were 10 years or more in practice were more likely to report regular ADHD care [44]. Family physician surveys in Ontario and British Columbia have indicated that most primary care physicians referred patients to a specialist for the diagnosis and management of ADHD [16, 45–47]. Also, about 80% of family physicians in an Ontario survey responded that they wanted additional training in the management of patients with ADHD [46] and this finding is consistent with more recent Canadian studies [16, 47, 48]. The increased intensity of patient visits for ADHD during the COVID-19 pandemic has highlighted the need for primary care physicians to have the necessary mental health supports in place to assist in the diagnosis and management of these patients.

While family physicians are the gatekeepers of health care in Ontario, Canada for patients seeking ADHD services, family physicians are facing challenges in accurately identifying, diagnosing, and managing ADHD in the primary care setting. Individuals with ADHD require more specialized care pathways beyond what is typically available in a family physician’s practice. This means that greater support and resources may be needed in primary care to support all family physicians in providing the comprehensive care that is needed to individuals with ADHD across all ages. Such care should be readily accessible, timely and integrated with other health care professionals such as psychiatrists, psychologists, behaviour health specialists, nurses and even schools. More focus on integrated care models in primary care may be needed so that family physicians can become better equipped to handle the demands of increased health care services for ADHD. Future research on the delivery of integrated health care services in primary care to meet the demands of individuals with ADHD should be explored.

This study has certain strengths. It used EMR data from a large primary care database in Ontario to examine ADHD medication trends in ages 5–55 years prior to and during the pandemic, a feature that is unique to the UTOPIAN Data Safe Haven database. Other provincial health administrative databases for medications in Canada may not have information about prescriptions for all age groups. This study also provided data on the pandemic period which spanned from March 2020 to December 2021, capturing a total period of 22 months where other published studies have reported on the immediate impact of the pandemic over a much shorter time [12, 13, 15, 42, 49]. This study also has limitations. Only patient visits with UTOPIAN family physicians were considered in this study representing a convenience sample of primary care providers in the Greater Toronto Area and beyond and therefore may not reflect the situation in Ontario. UTOPIAN contributors are primarily from academic family health teams and include fewer family physicians with independent community practices. Furthermore, patients with ADHD-related visits may have sought care from other health care providers and/or private uninsured services without accessing their family physicians first. Seeking such care would not have been measured in this study thereby reflecting an underestimation of the actual demand for insured mental health services for ADHD in the primary care setting during the COVID-19 pandemic. When billing for a visit with a patient who has ADHD, family physicians may have chosen other diagnostic codes to describe the primary reason for the visit such as the OHIP billing codes 313 or 300. Although the measures used in this study are highly specific to ADHD care, they do not necessarily reflect the full scope of primary care provided to patients with ADHD.

## Conclusion

We saw an increased demand on family physicians to provide mental health services for those presenting with ADHD-related visits during the second year of the COVID-19 pandemic. While primary care is the first point of access for most patients seeking ADHD care in Ontario, Canada, family physicians were assessing patients for ADHD-related visits more frequently than before the COVID pandemic. Given the significant degree of impairment on academic, social, and/or occupational functions that patients presenting with ADHD face across the ages coupled with the trends that we observed for increased visits to primary care physicians as we continue living with the pandemic suggest that resources to support family physicians may be needed.

## Supporting information

S1 Appendix List of OHIP service codes. (RTF)

S1 Table List of ADHD specific medications. (DOCX)

S2 Table Data for Figs 1–3. (DOCX)

S3 Table Observed and expected number of ADHD-related visits. (DOCX)
