# Supplementary material for: Patient visits and prescriptions for attention-deficit/hyperactivity disorder from 2017–2021: Impacts of COVID-19 pandemic in primary care
Source: PLoS One. 2023 Mar 13;18(3):e0281307. doi: 10.1371/journal.pone.0281307 (PMC10010552; doi:10.1371/journal.pone.0281307)
Supplement: S1 Appendix — (RTF) [file pone.0281307.s001.rtf]

S1 Appendix: List of OHIP service codes
We used the following OHIP service codes to describe a visit to a primary care physician:
A001|A002|A003|A004|A007|A008|A071|A131|A134|A624|A888|A903|A920|K005|K007|K013|K017|K022|K028|K030|K032|K033|K039|K130|K131|K132|K680|P003|P004|P005|P008|K037|K080|K081|K082|K087|K088|K089
Note: an ADHD visit was defined when one of these OHIP service codes was recorded with the diagnostic ICD-9 code of 314. 
