# Supplementary material for: Patient visits and prescriptions for attention-deficit/hyperactivity disorder from 2017–2021: Impacts of COVID-19 pandemic in primary care
Source: PLoS One. 2023 Mar 13;18(3):e0281307. doi: 10.1371/journal.pone.0281307 (PMC10010552; doi:10.1371/journal.pone.0281307)
Supplement: S1 Table — (DOCX) [file pone.0281307.s002.docx]

S1 Table: List of ADHD specific medications

| **Generic** |
| --- |
| methylphenidate |
| lisdexamfetamine |
| amphetamine amfetamine |
| dextroamphetamine dexamfetamine  dextroamfetamine |
| atomoxetine |
| guanfacine |
| modafinil |
