# Supplementary material for: Patient visits and prescriptions for attention-deficit/hyperactivity disorder from 2017–2021: Impacts of COVID-19 pandemic in primary care
Source: PLoS One. 2023 Mar 13;18(3):e0281307. doi: 10.1371/journal.pone.0281307 (PMC10010552; doi:10.1371/journal.pone.0281307)
Supplement: S2 Table — (DOCX) [file pone.0281307.s003.docx]

**S2 Tables: Data for Figures 1, 2 and 3**

**S2A Table: Data for expected and observed trends in ADHD visit rates (per 1000 patients) for Figure 1**

| **Year** | **Observed rate (per 1000 patients)** | **Expected rate (per 1000 patients)** | **Lower expected rate (per 1000 patients)** | **Upper expected rate (per 1000 patients)** | **Z-statistic** | **P-value** |
| --- | --- | --- | --- | --- | --- | --- |
| 2017 | 5.77 | 5.94 | 4.75 | 7.13 | -0.28 | 0.78 |
| 2018 | 7.28 | 6.97 | 5.55 | 8.40 | 0.43 | 0.67 |
| 2019 | 8.14 | 8.30 | 6.54 | 10.05 | -0.18 | 0.86 |
| 2020 | 11.38 | 9.93 | 7.76 | 12.11 | 1.30 | 0.19 |
| 2021 | 16.36 | 11.94 | 9.22 | 14.66 | 3.19 | 0.001 |

**S2B Table: Data for expected and observed trends in ADHD visit prevalence (per 1000 patients) for Figure 2**

| **Year** | **Observed rate (per 1000 patients)** | **Expected rate (per 1000 patients)** | **Lower expected rate (per 1000 patients)** | **Upper expected rate (per 1000 patients)** | **Z-statistic** | **P-value** |
| --- | --- | --- | --- | --- | --- | --- |
| 2017 | 3.66 | 3.73 | 3.00 | 4.46 | -0.18 | 0.86 |
| 2018 | 4.53 | 4.40 | 3.53 | 5.27 | 0.28 | 0.78 |
| 2019 | 5.20 | 5.26 | 4.19 | 6.34 | -0.12 | 0.91 |
| 2020 | 6.01 | 6.34 | 5.00 | 7.68 | -0.47 | 0.64 |
| 2021 | 8.14 | 7.65 | 5.97 | 9.33 | 0.57 | 0.57 |

**S2C Table: Data for expected and observed trends in ADHD prescription prevalence (per 1000 patients) for Figure 3**

| **Year** | **Observed rate (per 1000 patients)** | **Expected rate (per 1000 patients)** | **Lower expected rate (per 1000 patients)** | **Upper expected rate (per 1000 patients)** | **Z-statistic** | **P-value** |
| --- | --- | --- | --- | --- | --- | --- |
| 2017 | 12.53 | 12.42 | 9.83 | 15.02 | 0.08 | 0.93 |
| 2018 | 13.86 | 14.04 | 11.09 | 16.98 | -0.12 | 0.90 |
| 2019 | 16.03 | 15.93 | 12.54 | 19.31 | 0.06 | 0.95 |
| 2020 | 17.13 | 18.06 | 14.18 | 21.94 | -0.47 | 0.64 |
| 2021 | 21.30 | 20.39 | 15.97 | 24.81 | 0.40 | 0.69 |
