# Supplementary material for: Patient visits and prescriptions for attention-deficit/hyperactivity disorder from 2017–2021: Impacts of COVID-19 pandemic in primary care
Source: PLoS One. 2023 Mar 13;18(3):e0281307. doi: 10.1371/journal.pone.0281307 (PMC10010552; doi:10.1371/journal.pone.0281307)
Supplement: S3 Table — (DOCX) [file pone.0281307.s004.docx]

**S3 Table: Observed and expected number of ADHD-related visits**

| **Year** | **Age Group** | **Sex** | **Observed** | **Expected [95% CI]** | **Ratio [95% CI]** |
| --- | --- | --- | --- | --- | --- |
| 2020 | 05-09 years | Female | 29 | 28 [8, 47] | 1.05 [0.61, 3.60] |
| 2020 | 10-14 years | Female | 57 | 65 [13, 117] | 0.88 [0.49, 4.23] |
| 2020 | 15-19 years | Female | 132 | 104 [10, 197] | 1.28 [0.67, 12.58] |
| 2020 | 20-24 years | Female | 110 | 34 [6, 61] | **3.27 [1.79, 18.63]** |
| 2020 | 25-34 years | Female | 141 | 102 [28, 176] | 1.39 [0.80, 5.10] |
| 2020 | 35-55 years | Female | 113 | 89 [25, 154] | 1.26 [0.73, 4.54] |
| 2020 | 05-09 years | Male | 144 | 174 [64, 284] | 0.83 [0.51, 2.26] |
| 2020 | 10-14 years | Male | 132 | 151 [27, 275] | 0.87 [0.48, 4.94] |
| 2020 | 15-19 years | Male | 202 | 229 [2, 456] | 0.88 [0.44, 113.10] |
| 2020 | 20-24 years | Male | 183 | 171 [54, 289] | 1.07 [0.63, 3.40] |
| 2020 | 25-34 years | Male | 243 | 131 [37, 225] | **1.85 [1.08, 6.62]** |
| 2020 | 35-55 years | Male | 160 | 165 [13, 318] | 0.97 [0.50, 12.17] |
| 2021 | 05-09 years | Female | 64 | 25 [7, 42] | **2.6 [1.53, 8.85]** |
| 2021 | 10-14 years | Female | 87 | 81 [17, 145] | 1.07 [0.60, 5.09] |
| 2021 | 15-19 years | Female | 200 | 128 [15, 242] | 1.56 [0.83, 13.75] |
| 2021 | 20-24 years | Female | 166 | 27 [4, 50] | **6.08 [3.3, 38.27]** |
| 2021 | 25-34 years | Female | 243 | 125 [34, 216] | **1.94 [1.12, 7.18]** |
| 2021 | 35-55 years | Female | 197 | 102 [28, 175] | **1.93 [1.12, 6.92]** |
| 2021 | 05-09 years | Male | 139 | 197 [73, 321] | 0.70 [0.43, 1.90] |
| 2021 | 10-14 years | Male | 147 | 178 [30, 326] | 0.83 [0.45, 4.95] |
| 2021 | 15-19 years | Male | 254 | 287 [3, 571] | 0.88 [0.44, 94.18] |
| 2021 | 20-24 years | Male | 262 | 229 [72, 385] | 1.15 [0.68, 3.66] |
| 2021 | 25-34 years | Male | 284 | 144 [39, 249] | **1.97 [1.14, 7.21]** |
| 2021 | 35-55 years | Male | 222 | 232 [18, 447] | 0.95 [0.50, 12.14] |
